# Detection of somatic structural variants from short-read next-generation sequencing data

**DOI:** 10.1093/bib/bbaa056

**Published:** 2020-05-07

**Authors:** Tingting Gong, Vanessa M Hayes, Eva K F Chan

**Keywords:** structural variant, cancer genomic, next-generation sequencing, variant caller

## Abstract

Somatic structural variants (SVs), which are variants that typically impact >50 nucleotides, play a significant role in cancer development and evolution but are notoriously more difficult to detect than small variants from short-read next-generation sequencing (NGS) data. This is due to a combination of challenges attributed to the purity of tumour samples, tumour heterogeneity, limitations of short-read information from NGS and sequence alignment ambiguities. In spite of active development of SV detection tools (callers) over the past few years, each method has inherent advantages and limitations. In this review, we highlight some of the important factors affecting somatic SV detection and compared the performance of seven commonly used SV callers. In particular, we focus on the extent of change in sensitivity and precision for detecting different SV types and size ranges from samples with differing variant allele frequencies and sequencing depths of coverage. We highlight the reasons for why some SV callers perform well in some settings but not others, allowing our evaluation findings to be extended beyond the seven SV callers examined in this paper. As the importance of large SVs become increasingly recognized in cancer genomics, this paper provides a timely review on some of the most impactful factors influencing somatic SV detection that should be considered when choosing SV callers.

## Introduction

Cancer is a disease of the genome that develops through the accumulation of somatic mutations (variants), ranging from single-nucleotide variants (SNVs), insertions/deletions (indels) of a few nucleotides, to copy number variations (CNVs) and large structural variants (SVs) [1]. SVs are typically defined as genomic rearrangements involving at least 50 nucleotide bases (50 bp) and broadly include deletions, insertions, duplications, inversions and translocations [2]. SV formation can leave complex genomic patterns, some of which are associated with specific cancer types while others are more broadly implicated. For example, chromothripsis, involving hundreds of clustered rearrangements arising from the shattering and inaccurate reassembly of a single chromosome, is found in 3% of all cancer types [3]. In contrast, chromoplexy, involving complex coordinated chains of rearrangements, is almost exclusively found in prostate cancer [4]. Thus, the extent and types of somatic SVs can help characterize tumour types and provide insights into the mechanisms of oncogenesis. Additionally, SVs that underlie oncogene activation and tumour suppressor loss can potentially be targeted for therapy or used as prognostic markers [1].

Due to continued reductions in cost and increase in throughput, next-generation sequencing (NGS) has become the preferred approach for cancer genomics [1, 5]. NGS, currently dominated by Illumina pair-end short-read sequencing, is a technology that allows the entire human genome to be read. Conceptually, the approach is simple: DNA from multiple cells is extracted and fragmented to a desired library size (typically 200–500 bp), then the ends of each fragment are tagged (so they can be tracked and paired) and sequenced inwards to up to ~150 bp [6]. Following sequencing, the billions of reads are informatically paired and aligned to a known reference genome for variant detection. SVs are inferred from abnormal alignment patterns suggestive of genomic rearrangement breakpoints. The underlying bioinformatic analyses are not straightforward for several reasons. Detection of SVs that are kilobases to megabases in length is difficult from short-sequence reads and small-insert library sizes (distance between pairs of reads) as they cannot be captured by any single sequence [7]. While sequencing of more reads (higher depth of coverage) can sometimes compensate for this, it provides limited advantage at genomic regions with low sequencing complexity (e.g. repetitive sequences) or regions of high sequence similarity (e.g. segmental-duplicated regions). These regions can lead to ambiguous read alignments, which are a significant source of false-positive (FP) variant detection.

Compared with germline SVs, the detection of somatic SVs in cancer genome (identification of SVs present in the cancer sample but absent in the patient matched-normal sample) is further complicated by tumour purity (fraction of cancerous to normal cells) and tumour heterogeneity (presence of clonal and subclonal tumour cell populations). Compounding onto this is that the extents of both of these confounders are typically unknown at the time of tissue sampling. Again, while increasing sequencing coverage can assist in capturing low abundance tumour SVs, in many cases, it is unclear whether the associated increase in cost can outweigh any information gained [7].

These challenges have resulted in the development and refinement of multiple SV detection methods and SV calling software (SV callers) in the past decade, each with their advantages and disadvantages. While studies have explored some of these effects on SV detection, many have focused on germline SVs [8, 9]. The most comprehensive study with focus on somatic SV detection compared the performance of 13 SV callers on three sets of simulated data [10]. They observed that some variables generated similar error profiles across SV callers but did not delve into the extent of these correlations.

In this paper, we provide a comprehensive review on common factors affecting the detection of somatic SVs and on the performance of seven commonly used SV callers. Specifically, we evaluate and quantify each SV caller’s ability to detect different SV types and size ranges, the individual and interaction effects of SV abundance and sequencing coverages, their precision in predicting genomic breakpoints as well as the impact of sequence similarity [genomic segmental duplications (SegDup)], sequencing biases (GC-content bias and homopolymers) and read-alignment quality on somatic SV detection.

## Methods

To objectively assess the impact of each parameter [SV type, SV size, variant allele fraction (VAF), sequencing coverage, sequence similarity] on different SV callers, we used a simulation framework detailed in Supplementary Data (available online at https://academic.oup.com/bib). In brief, for each evaluation setting (reviewed in the following sections), three replicate pairs of normal and tumour genomes were simulated to contain germline-only and germline plus cancer SVs, respectively, based on the human reference genome sequence GRCh38. Each simulated SV contains 1200 SVs including 200 of each of 6 SV types as described in the ‘Structural variant types and definitions’ section. Paired-end short-read sequences were then sampled from the augmented genomes using SVEngine [11] to the desired coverage. SV detection was performed following standard read-alignment against GRCh38 using BWA-MEM v0.7.17-r1194 [12]. Sensitivity and precision of SV callsets were evaluated based on two true-positive (TP) criteria: (1) the SV type reported for a candidate SV must match the simulated SV and (2) the genomic position of the reported breakpoints must be within a predefined distance from the simulated SV. Unless otherwise stated, evaluation results presented in this study are based on the default breakpoint resolution threshold of 200 bp as used in similar studies [8, 9].

## Results

### SV detection methods and callers

There are four main methods for the detection of SVs from short-read NGS data (Table 1): read-pair, read-depth, split-read and local-assembly [13]. Each of these methods is reliant on prealignment of sequencing reads to a reference genome.

**Table 1:** SV detection methods and example SV callers

Method	Detection resolution	Detectable SV types	Detectable SV sizes	Example SV callers	References
Read-pair	Rough	All	Median size SV	BreakDancer	Chen *et al.* [14]
Split-read	Base pair	All	Small size SV	Pindel	Ye *et al.* [24]
Read-pair and split-read	Base pair	All	Depend on filtering/scoring	Delly	Rausch *et al.* [29]
				Lumpy	Layer *et al.* [30]
Read-pair, split-read and local-assembly	Base pair	All	Depend on filtering/scoring	Manta	Chen *et al.* [33]
				GRIDSS	Cameron *et al.* [26]
				SvABA	Wala *et al.* [27]

The read-pair method searches discordant alignment signatures of paired-end sequencing reads. SVs are identified from read-pairs whose mapped interval is different from the sequencing library insert size or mapped in abnormal orientation. Compared with other methods (such as split-read), read-pair is less sensitive to small indels, especially SVs smaller than the library insert size [2]. BreakDancer is an example SV caller that uses the read-pair method [14].

The read-depth method seeks changes in the amount of sequencing coverage at a given genomic interval (segment window) relative to neighbouring or genome-wide coverage. It is particularly not only suited for the detection of CNVs but also capable of detecting some SV types with associated copy number changes, including deletion, duplication and insertion events. A significant difference between CNV and these SV types is that the latter are agnostic to the actual number of DNA copies gained or lost. As such, using only this method for SV detection can result in poor breakpoint resolution, which is also heavily dependent on overall depth of coverage and segment window size. Therefore, CNVs and SVs are generally treated as separate classes of variants. This is reflected in the numerous tools developed solely for the purpose of CNV detection [15–17]. Similarly, SV callers rarely attempt to infer copy number changes. Many studies have reviewed and compared the underlying methods and associated tools for detecting CNVs [18–23]; thus, SV callers based only on the read-depth method is not included in this study.

The split-read method assesses continuity and completeness of NGS read alignments against a reference genome. Discontinuous and incomplete read alignments are indicative of SV events, from which breakpoints can be inferred to single-nucleotide resolution. However, while an incomplete alignment may signal the presence of a rearrangement breakpoint, the unaligned portion provides little information on the adjacent sequence and not the type and size of the SV. This is particularly problematic for large insertions—a distinct disadvantage from not using paired-end information. In addition, genomic regions with high sequence similarity can confound the split-read method, and high sequencing depth is often needed to obtain sufficient split-reads overlapping the breakpoint to achieve a confident SV call. Pindel is an example of (and the first) SV caller to use the split-read method; it was one of the tools used to generate the callset in the 1000 Genome Project [24, 25].

The local-assembly method attempts to reconstruct rearranged genomic sequences by assembling sequencing reads associated with SV breakpoints as determined from the initial read-alignment. Therefore, local-assembly is usually implemented along with read-pair and split-read methods. Significant advantages of this approach are its ability to detect SVs at genomic regions with higher levels of sequence identity (non-uniqueness) and its ability to reconstruct novel inserted sequences and small highly rearranged sequences. However, a key disadvantage is the requirement for sufficient variant reads for reliable consensus contig assembly; a potential problem for cancer genomes where variants may only be present at low levels. GRIDSS [26] and SvABA [27] are example SV callers utilizing genome-wide local-assembly.

Aiming to overcome inherent limitations and to take advantage of the different approaches, a number of SV callers have incorporated multiple methods. For example, inGAP-SV [28] incorporates both read-pair and read-depth methods and has been shown to perform better than callers based only on read-pair method for germline SV detection [8]. Delly [29] and Lumpy [30] call SVs using both discordant paired-end and split-read alignments. Delly predicts SVs using discordant paired-end reads then use split-reads to refine SV breakpoints. In contrast, Lumpy integrates multiple alignment signatures into a single SV discovery process. In addition to paired-end and split-read alignment signatures, TIDDIT [31] and SVelter [32] further incorporate read-depth, whereas Manta [33], GRIDSS and SvABA further incorporate local-assembly to improve SV detection. Manta assembles reads in candidate SV regions identified from discordant paired-end and split-read alignments (i.e. targeted assembly), to validate and refine breakpoints, whereas GRIDSS and SvABA assemble all aberrantly aligned reads to identify breakpoints. SvABA applies assembly in local 25 kbp assembly windows, called windowed local-assembly, whereas GRIDSS performs assembly of all reads aligned improperly and terms it genome-wide break end assembly.

There are currently hundreds of SV callers available for SV detection from NGS data. Most, including inGAP-SV, TIDDIT and SVelter, are mainly designed for the detection of germline SVs, which are variants relative to the genome reference. In contrast, the identification of somatic SVs must exclude those observed in both the tumour and matched-normal samples as they represent either germline SVs (hence not relevant to the cancer genome) or are reference artefacts [34]. Most somatic SV callers perform two-sample (matched tumour–normal pair) variant calling and require further manual filtering (typically by the user) for SV calls present only in the tumour sample (e.g. BreakDancer, Lumpy, GRIDSS). For some SV callers, such as Manta, Delly and SvABA, the filtering step is automated.

In general, SV callers leveraging multiple detection methods have the best balance between sensitivity and precision for the detection of germline SVs, though there are notable differences in their performance for different SV types and sizes [8, 9]. For somatic SVs, the recent ICGC-TCGA DREAM Somatic Mutation Calling Challenge, which evaluated the performance of 13 SV callers, found the overall sensitivity and precision of somatic SV calling to be highly influenced by lower allelic fractions of subclonal variants, tumour sequencing depth and read-alignment quality at SV breakpoints [10]. However, this study did not include other important factors such as SV types and sizes.

#### SV callers based on multiple methods are more accurate

**Figure 1:**
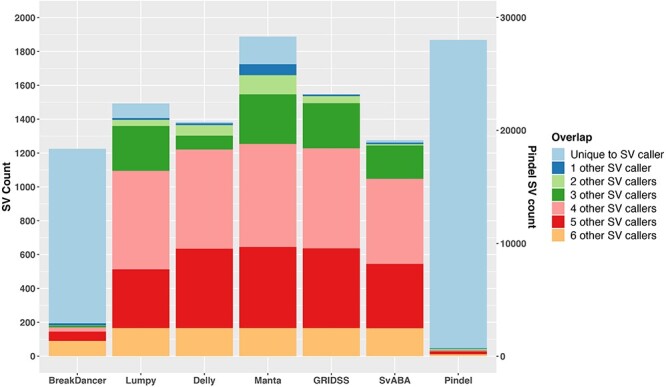
Extent of SVs identified by different callers and their overlaps. Results are based on simulation data with tumour and matched-normal coverage of 60× and VAF of 100%. The count of Pindel is labelled on the right vertical axis.

**Figure 2:**
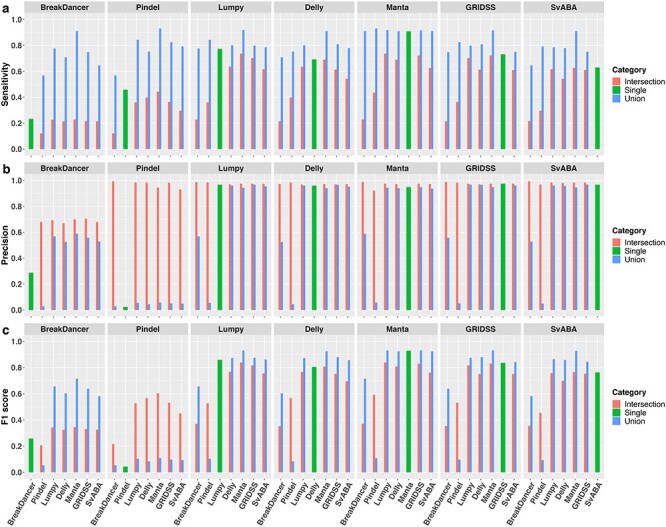
Overall sensitivity, precision and F1 score of individual SV callers and their pairwise union and intersection callsets. Results are based on simulation data with tumour and matched-normal coverage of 60×, VAF of 100% and breakpoints within 200 bp of simulated SVs.

In this section, we compare the overall performance of the seven SV callers representing the common SV detecting methods (Table 1). As the SV callers are reliant on different detection methods, their predicted SVs are rarely completely concordant and the total number of somatic SV calls can be widely different (Figure 1). As previously noted, Pindel is very sensitive to split-read signatures, thus the majority (97.7%) of detected variants are <50 bp, which are typically considered as small indels rather than large SVs. For the purpose of this study, variants <50 bp detected by Pindel were excluded. Overall, Pindel and BreakDancer, which are based on a single SV detection method, detect many unique calls, not identified by other callers. In contrast, SV callers, based on at least two SV detection methods, are more concordant. Comparing their overall performance (Figure 2, green bars), we confirmed SV callers based on more than one SV detection methods have higher sensitivity and lower false discovery rate, similar to observations for germline SV detection [8, 9]. Additionally, these SV callers (Manta, Lumpy, GRIDSS, SvAVA and Delly) were observed to have higher precision (>90%) than sensitivity, ranging from 63% (SVABA) to 91% (Manta).

#### Inclusion of additional callers to an already high-performing SV caller provides little gain to sensitivity and precision

Due to inherent limitations of each SV caller, it is common practice to use at least two SV callers [13] in order to maximize detection sensitivity and precision. In general, a union callset from a pair of SV callers will be more sensitive than either of the single SV callers alone but at the cost of reduced precision, whereas an intersection callset will improve precision at the cost of reduced sensitivity. To evaluate the extent of gain when combining SV callers, we compared the performance of all pairs of SV callers for both union and intersection callsets. As expected, union callsets (Figure 2, blue) greatly improve sensitivity (>14%), compared with individual SV callers, with the exception of Manta, which has at most only 2% increase when combined with Pindel. However, the impact of union callsets on precision is more variable. When coupled with an ‘imprecise’ SV caller (precision <30% as a standalone SV caller), such as BreakDancer or Pindel, precision of the union callsets can be worsen by up to 94%. In contrast, the union callsets of two similarly ‘precise’ SV callers (precision >90%) typically have little impact on precision (<3%) compared with using just a single caller. As expected, we observed dramatic improvement in precision when the callset of an imprecise SV caller is intersected with a precise caller, whereas less improvement (<10%) is observed when both callers in the pair are already ‘precise’. In contrast, changes in sensitivity of intersection callsets are more variable. For example, the intersection callsets between Manta and Lumpy or BreakDancer improved precision by 3 and 2% respectively, compared with using Manta alone, but reduce sensitivity by 17 and 55%, respectively. Overall, Manta achieved the highest F1 score among individual SV callers and, at most, only about 1% increase in F1 score was gained through its union callsets with Lumpy or GRIDSS (Figure 2**C**).

#### Better performing SV callers do not require longer runtime

**Figure 3:**
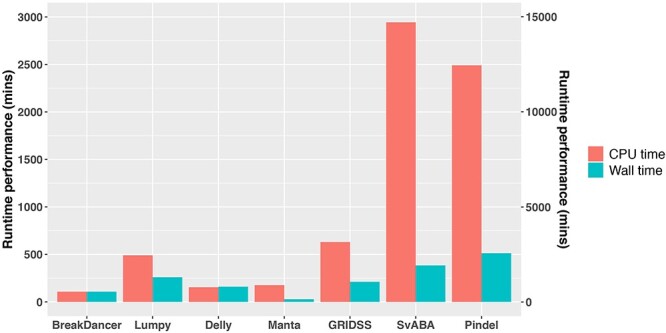
Runtime performance of SV callers. Results are based on simulation data with tumour and matched-normal coverage of 60× and VAF of 100%. Total CPU time indicates total CPU utilization in parallel-processing mode. Wall time indicates real total elapsed time and can be less than total CPU time if the caller can be efficiently multithreaded. The wall and CPU time of Pindel is labelled on the right vertical axis. Runtime performance was measured in parallel-processing mode using eight cores on a dual socket Xeon E5-2680 V3 server.

In addition to overall performance in somatic SV detection accuracy, the amount of computational resources required to run any bioinformatics software can pose logistical limitations. As such, we also compared the computational efficiency, including wall time and total central processing unit (CPU) hours for each of the seven SV callers (Figure 3). Pindel was found to have the longest runtime, as previously observed [8, 9]. The preprocessing step of Lumpy using SAMTOOLS (Supplementary Data available online at https://academic.oup.com/bib) was found to require a long runtime. Wall time and CPU time were similar for BreakDancer and Delly as both tools utilize only a single core. In contrast, SvABA and Manta are able to efficiently use all cores assigned, thus substantially reducing wall time. In addition, SV callers incorporating local-assembly typically require more CPU time, though Manta appears to be an exception.

### SV types and definitions

**Figure 4:**
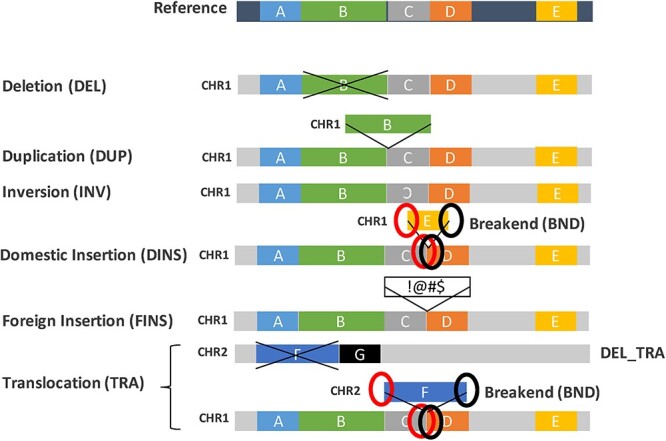
Definition of SV types. A ‘normal’ representation of the reference genome (dark blue background) with five schematic regions (labelled **A**–**E**) is shown at the top. Below are different representations of rearranged genomes (grey background), relative to the reference, corresponding to each of the SV types: DEL, DUP, INV, INS (DINS or FINS) and TRA. DINS and TRA can be represented by two pairs of BND.

Within a cancer, multiple genomic rearrangements could occur simultaneously or in series and could occur in a subset of cells or multiple clones, giving raise to complex SV signatures. For simplicity, SVs are broadly classified into six types in this study (Figure 4). Deletion (DEL) is the removal of a DNA segment from the genome. Duplication (DUP), also known as tandem duplication, is the event of copying a DNA segment and inserting it beside the original copy. Inversion (INV) is the inversion of a DNA segment at the same locus. Insertion (INS) has two subtypes: domestic insertion (DINS) is the addition of a DNA segment copied from a distant site of the same genome (i.e. ‘copy-and-paste’) and foreign insertion (FINS) is the addition of a novel sequence, not known to be present in the sample genome. Translocation (TRA) involves the deletion of a DNA segment from one locus and its reinsertion at another locus (i.e. ‘cut-and-paste’). As a result, a translocation event is associated with a deletion event (DEL_TRA). It is worth noting here that, while a DEL event will be accompanied by a copy number loss, a DEL_TRA is copy-number neutral from a CNV perspective. Furthermore, in accordance with the Variant Call Format (VCF) 4.2 specification (updated 8 March 2019), a fusion junction in a rearranged genome can further be described by two adjacent break ends (BND) with coordinates relative to the reference genome, such that DINS and TRAs can be represented by two pairs of BNDs (Figure 4).

In spite of these broad classes, operational definitions for each SV category can vary between SV callers (Table 2, Supplementary Data available online at https://academic.oup.com/bib). BreakDancer can detect and reports DEL, INV, INS, which could include DINS, FINS and TRA), intra-chromosomal translocation (ITX, which could include DUP and DINS) and inter-chromosomal translocation (CTX, which could include DINS). Pindel reports DEL, DUP, INV, INS and replacement (RPL). A RPL describes an insertion event around the breakpoint of a deletion event, which could in fact capture DUP, INV, DINS, FINS and TRA, thus occasionally resulting in duplicate SV calls with different assigned SV types. Lumpy and Delly both based on both read-pair and split-read alignment signatures can detect DEL, DUP, INV and BND of inter-chromosomal events irrespective of copy-and-paste or cut-and-paste, thus could include both DINS and TRA. Delly detects small INS that can be captured by single reads (smaller than read-length), whereas Lumpy cannot detect small INS. Neither can detect full intra/inter-chromosomal insertions with insert fragments exceeding the library insert size; evidence of (unpaired) breakpoints for these events is reported as BND. Manta can detect and report DEL, DUP, INV, INS (fully and partially assembled insertions) and BND of inter-chromosomal events. GRIDSS being an SV breakpoint caller initially reports all variants as BND but can be post-annotated as DEL, DUP, INV, INS and BND with its accompanying R script. SvABA, similar to GRIDSS, initially reports all SVs as BND with SV types further assigned, where possible, according to breakpoint orientations, as DEL, INV, and DUP or INS (DUP/INS), which cannot be distinguished through breakpoint orientation.

**Table 2:** Comparison of SV types and definitions across SV callers

Broad SV type	Break Dancer	Pindel	Lumpy	Delly	Manta	GRIDSS	SvABA
Deletion	DEL	DEL	DEL	DEL	DEL	DEL	DEL
Duplication	ITX	DUP, RPL	DUP	DUP	DUP	DUP	DUP/INS
Inversion	INV	INV, RPL	INV	INV	INV	INV	INV
Domestic insertion	INS, ITX, CTX	INS, RPL	BND	INS, BND	INS, BND	INS, BND	DUP/INS, BND
Foreign insertion	INS	INS, RPL	n/a	INS	INS	INS	DUP/INS
Translocation	INS, ITX, CTX	INS, RPL	BND	INS, BND	INS, BND	INS, BND	DUP/INS, BND

*Note*. n/a: The SV type cannot be detected by the corresponding SV caller.

**Figure 5:**
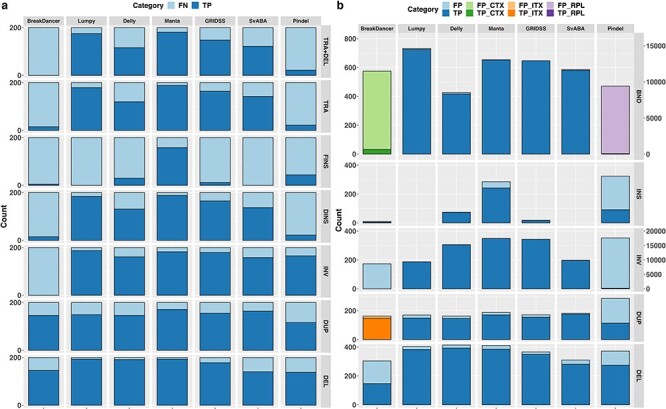
Performance of SV callers in detecting different SV types. (**A**) The number of simulated SVs recovered (dark blue; TP) and missed (light blue; FN) by individual SV callers for each SV category: DEL, DUP, INV, DINS, FINS, TRA and TRA + DEL detected. (**B**) The total number of SVs reported by individual SV callers that are TPs (dark shade) or FPs (light shade). The total number of DUPs shown under SvABA can be either DUP or INS events. The number of CTX reported by BreakDancer is shown in the BND category in green. The number of ITX reported by BreakDancer is shown in the DUP category in orange. The number of RPL reported by Pindel is shown in the BND category in purple. Counts of RPL and INV by Pindel are labelled on the right.

Here, we evaluate each SV caller’s ability and efficiency to detect different SV types using a set of simulated somatic SVs comprising of 200 of each of the six SV types (Table 2 and Supplementary Data available online at https://academic.oup.com/bib for definition of concordance).

#### DELs are the easiest to detect, whereas FINS are the most difficult

Overall, all SV callers have highest sensitivity and precision for detecting DEL compared with other SV types. BreakDancer performs modestly for DEL events with 73% recall rate but 52% FP rate. It should be noted that the DEL counts shown in Figure 5**B** include both DEL and DEL_TRA. Thus, in fact, the high FP rate of DEL calls from BreakDancer were due to its limitation in detecting DEL_TRA with precise breakpoint (typically >200 bp). Interestingly, while Pindel has overall recall rate of only 45% (Figure 2**A**), it exceeds this for two subclasses of SVs, recovering 69% DEL and 83% INV (Figure 5**A**). However, Pindel is overly sensitive in detecting INV and RPL. Of >28 000 reported somatic SV calls, 63% are INV and 34% are RPL calls, and almost all are FPs (Figure 5**B**).

Unlike the other SV callers, BreakDancer has extremely low sensitivity for detecting INV, whereas Pindel has a high FP rate. It is worth noting here that, while Manta, GRIDSS and Delly reported more INV than were simulated (Figure 5**B**), they do not have proportionately higher FPs. This is because these three SV callers report two paired-end clusters for each INV (INV3 and INV5 tags for Manta and CT = 3 to 3 or 5 to 5 tags for Delly in VCF/INFO field and 2 pairs of BND for GRIDSS). Taking this into consideration, the three SV callers are able to recover >80% of INV while missing <10% (Figure 5**A**).

All SV callers struggled to detect simulated FINS, with 79% detected by Manta and <30% recovered by other SV callers (Figure 5**A**). Of the FINS recovered by the SV callers with the exception of Manta, all were <500 bp, which is the mean insert size simulated. This poor performance in FINS detection is from all callers being reliant on the initial alignment of sequencing reads to the reference genome. In case of FINS, where the inserted sequence is absent in the reference genome, the corresponding reads are typically missed early in the SV detection pipeline, or even filtered out prior to SV detection. Therefore, FINS can only be inferred from reads that align in a split-read manner or reads whose mates are properly aligned, resulting in only small FINS within the library insert size range can be detected. Differently, Manta reports large insertions even though the inserted sequence cannot be fully assembled, resulting in higher recall rate in FINS detection. However, Manta had 15% FP INS calls, significantly higher than other SV callers (Figure 5**B**). Interestingly, those FP INS calls were small (<102 bp) and reported with incorrect SV types.

#### Complex genomic rearrangements can give raise to multiple SV signatures

Although TRA and DINS events were not explicitly reported, all SV callers perform well for these two SV types, except BreakDancer and Pindel. Pindel is able to report small (~50 bp) INS, including TRA and DINS events, but is unable to identify larger rearrangements including inter-chromosomal events. BreakDancer has limited ability in detecting discordant read-pairs aligned to different chromosomes, thus resulting in high FP CTX detection (94%; Figure 5**B** green)**.** This is in contrast to its high recall for ITX with the majority (91%) being true DUP events (Figure 5**B** orange; Table 2).

Typically, TRA are identified by BND signatures (rearrangement junction) or insertion signatures from NGS data. Using only this as a criterion, 60–94% TRA (Figure 5**A**) were recovered by the remaining five SV callers as they are able to detect BNDs, and insertion signatures of small TRA events (<100 bp), with high sensitivity and precision. However, because the TRA were simulated as cut-and-paste events in our study, DEL signatures associated with the TRA should also be present in the data. For >85% of the detected TRA signatures, their associated DEL signatures could also be found (Figure 5**A** top row), suggesting these SV callers can potentially subclassify translocation into those that are copy neutral and those with copy number gain. Similarly, while DINS were simulated as copy-and-paste events, many SV callers consider insertion and copy number gain signatures separately. In our data, 66–94% DINS were recoverable as having BND or an insertion signature (Figure 5**A**). However, as read-depth was not tightly integrated into these SV callers, it is unclear how well copy-and-paste events could be correctly identified. It is worthy of note that, while Lumpy has a read-depth option that takes into consideration of CNV estimates from auxiliary CNV callers for filtering and refining their SV estimates, copy number change is not incorporated in the algorithm *per se* for the detection of copy-and-paste events [30].

Although both Delly and Lumpy are based on a combination of read-pair and split-read alignment signatures, Delly missed many more TRA and DINS (40 and 34%, respectively) than Lumpy. This is because Lumpy integrates both alignment signatures for SV discovery, whereas Delly uses split-read only for SV breakpoint refinement. We note that all simulated intra-chromosomal DINS and 50% of intra-chromosomal TRA events were missed by all SV callers due predominantly to incorrectly reported SV types. For instance, an intra-chromosomal DINS is typically falsely reported as both a tandem DUP and a DEL, with reported SV intervals extending into the flanking regions of the simulated DINS.

#### SV callers based on multiple detection methods have similar overall performance profile across different SV types

In summary, SV callers based on more than one method in the discovery step have better overall performance across all SV types, except for FINS. As expected, DEL is the ‘easiest’ to recover. Interestingly, INV and TRA have similar recovery rates as DUP, suggesting the relatively fewer reported cases of INV and TRA in the literature is due to these two SV types being genuinely less prevalent in cancer genomes than DUPs, rather than it being a methodological limitation. Among the seven SV callers examined, Manta and Lumpy performed the best, with most notable differences in Manta being able to detect DUP better (7.6% higher F1 score) but Lumpy being able to detect BND better (5% higher F1 score; Supplementary Table S1 available online at https://academic.oup.com/bib).

### Impact of SV sizes on SV detection

**Figure 6:**
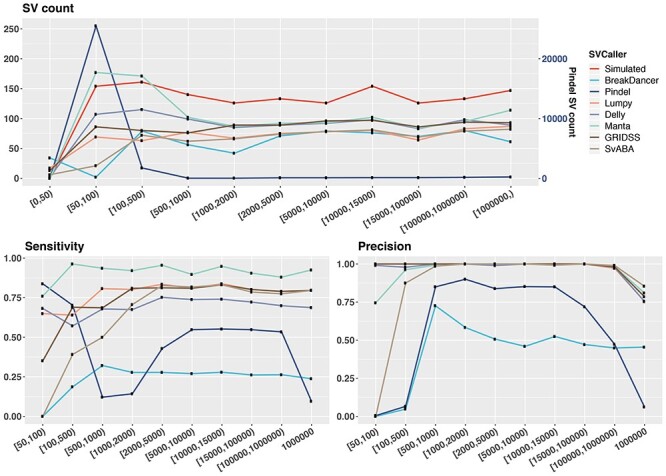
Performance of SV callers across different variant size ranges. Shown on top is the number of SVs reported by each SV caller within each defined SV size range. The bottom plots show sensitivity (left) and precision (right) evaluated based on a breakpoint precision threshold of 200 bp. Results are based on simulated samples with tumour and matched-normal coverage of 60× and tumour purity of 100%. Size of TRA corresponds to the size of the translocation DNA fragment. The Pindel SV count is on the right vertical axis.

SVs are distinguished from small indels by the number of bases they impact with 50 bp being the commonly accepted threshold for classification as SVs. Here, we compared the SV callers’ ability to detect SVs at different size ranges, from 50 bp to 1 Mbp. Most SV callers are consistent in the overall number of SVs detectable across different size ranges (Figure 6). We note here that, BreakDancer is unable to estimate the size of translocated DNA fragments and so assigns an arbitrary 498 bp for all reported CTX events; these are excluded in Figure 6.

All SV callers struggled to detect SVs <100 bp, with Pindel achieving highest sensitivity of 83%, which is still lower than the highest sensitivity for other size ranges. In particular, we note 14 DUPs were missed by all callers, of which 12 were small (50 bp). This was due to (1) an absence of discordant read-pair signature for SV callers that use this detection method (e.g. BreakDancer, GRIDSS), (2) alignment uncertainly for callers that do not perform realignment or assembly (e.g. Lumpy, Delly) and/or (3) small DUPs being misreported as INS calls by callers that use heuristic SV lengths for distinguishing between INS and tandem DUP (e.g. GRIDSS, Pindel). In general, most SV callers detect small SVs (<100 bp) with lower precision, with GRIDSS, Delly and Lumpy being the exceptions (>99%). Additionally, all SV callers have lower precision in detecting large SVs (1 Mbp), with SvABA having the highest precision at 85%. However, SvABA has zero sensitivity and precision in detecting SVs within 50–100 bp. In fact, the smallest SV size reported by SvABA is 97 bp; this is due to an arbitrary cut-off used by SvABA (taken from BWA-MEM) to distinguish SVs and indels. This likely explains its overall lower performance compared with other multi-method SV callers.

In conclusion, SV callers based on more than one SV detection methods show highest consistency in performance across the different SV size ranges. However, there are still limitations to accurately detect SVs >1 Mbp for all SV callers. The split-read based method shows its power in detecting smaller SVs (50–100 bp), though it needs to be well incorporated with other methods to attain a good performance in both sensitivity and precision.

### Breakpoint precision of SV calls

**Figure 7:**
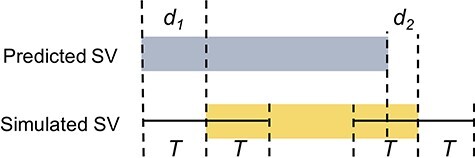
Breakpoint resolution threshold definition. A breakpoint resolution threshold, T, is defined as the absolute distance from a simulated breakpoint position within which a reported SV must fall. Thus, in this diagram, both *d*_1_ and *d*_2_ must be less than the defined T, for the predicted SV to be considered a TP call.

Breakpoint features are important for elucidating the underlying mechanisms of SVs, such as micro-homology-mediated DNA repair and movements of transposable elements [35]. Precise SV breakpoint positions can also facilitate accurate annotation of SVs, including impact on transcript splice sites and regulatory elements [25]. Furthermore, when comparing SV callsets, there is a need to define sufficient closeness between two calls for consideration as identical calls [10]. To better understand the extent of breakpoint resolution across the SV callers, we evaluate the change in the number of TP calls at varying breakpoint precision threshold from 0 to 2000 bp (Supplementary Data available online at https://academic.oup.com/bib). Breakpoint resolution is defined as the absolute distance between a true (simulated) breakpoint position and that reported by an SV caller (*d*_1_ and *d*_2_ in Figure 7).

#### SV callers using split-read method consistently detect SVs within 2 bp resolution

**Figure 8:**
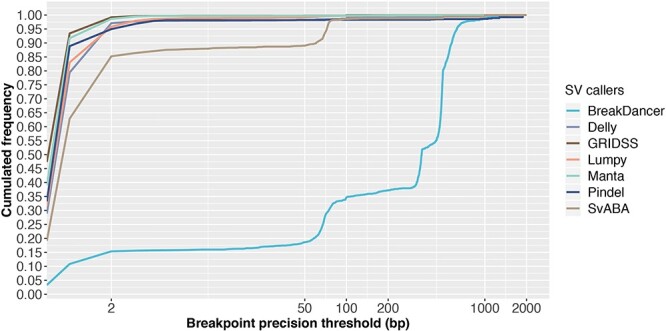
SV calling sensitivity at increasing breakpoint resolution threshold. Results are based on simulation data with tumour and matched-normal coverage of 60× and VAF of 100%. This figure shows the cumulated frequency of the number TP SVs detected by each caller for breakpoint precision thresholds <2000 bp.

BreakDancer has poor breakpoint resolution (Figure 8) requiring read-pairs to completely span rearrangement breakpoints and lie on either side of an SV event. Therefore, breakpoint locations can only be approximated by the mapping positions of discordant read-pairs; actual breakpoints are not captured. Pindel has great breakpoint precision with 95% of all SV calls within 2 bp resolution, reflecting the advantage of using split-read signatures. Again, SV callers using multiple SV detection methods have better overall breakpoint resolution, with 99% of breakpoints called within 2 bp for Manta and GRIDSS, within 5 bp for Lumpy and within 100 bp for SvABA.

While several SV callers are able to achieve near-perfect breakpoint resolution (within 2 bp), we observed a loss in precision with each additional processing step for all SV calling pipelines. In particular, we noted that between SV calling and SV annotation, reported breakpoints fluctuate by 1 or 2 bp, largely due to conversions between 0- and 1-based coordinates required for the different file formats (namely, BAM to VCF to BED and sometime back to VCF).

In addition, micro-homology around SV breakpoints can confound sequence alignment, which also contributes to imprecision of breakpoint prediction. To be mindful of micro-homology, users can check the confidence interval for breakpoint positions (CIPOS and CIEND) and base-pair identical micro-homology length and sequence (HOMLEN and HOMSEQ) in the output VCF files, as specified in the VCF 4.2 specification (updated 8 March 2019). However, of the seven SV callers examined in this study, only GRIDSS and Manta report all of these, whereas Lumpy only reports CIPOS and CIEND, and SvABA only provides HOMOLEN and HOMSEQ. In summary, SV callers based on more than one SV detection method have higher breakpoint detection resolution and split-read method is essential for precise breakpoint detection.

### Impact of VAF on SV detection

VAF is the proportion of sequencing reads supporting the detected variant at a given locus. In cancer genomics, two key factors influence VAF: tumour purity and tumour heterogeneity. Tumour purity is the proportion of cancerous cells captured in a sample, which is typically uncontrollable without the support of imaging-guided biopsy or histopathology-guided dissection. Tumour heterogeneity refers to the molecular diversity between cancer cells of a tumour sample and reflects the amount of genomic change throughput the development of the cancer. Obviously, SVs at low VAF are more difficult to detect than SVs in high abundance. However, detection of SV at low VAF can theoretically be compensated by increasing sequencing coverage [7]. In this review, we provide a comprehensive evaluation of the relationship and joint effect of VAF and sequencing coverage on SV detection from five SV callers, across different SV types and for different SV sizes. BreakDancer and Pindel were excluded in this analysis due to their notably poor performance even with 100% VAF as shown above (Figure 2) and previously by others [8].

#### Variant allele frequency has a non-linear impact on sensitivity and little impact on precision

**Figure 9:**
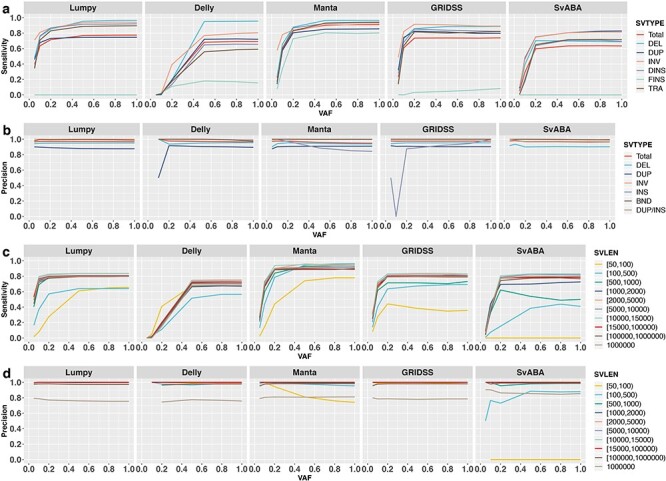
Impact of VAF on the detection of different SV types and sizes. Shown are sensitivities (**A** and **C**) and precisions (**B** and **D**) for different SV types (**A** and **B**) and SV size ranges (**C** and **D**) using five SV callers: Lumpy, Delly, Manta, GRIDSS and SvABA. Results are based on simulation data with tumour and matched-normal coverage of 60× and breakpoints within 200 bp of simulated SVs.

Overall, VAF has a monotonic but non-linear effect on sensitivity (Figure 9**A**, **C**) and little impact on precision (Figure 9**B**, **D**). The positive effect of VAF on sensitivity is greatest for lower VAF, with signs of saturation at VAF > 0.2 for all SV callers except Delly. While Delly appears to perform similarly to other SV callers at high VAF (>0.5), its performance is substantially worse at low VAF dropping to zero sensitivity at VAF < 0.1. In addition, at 0.1 VAF, Manta, Lumpy and GRIDSS maintain overall sensitivity at around 60%, whereas SvABA sensitivity drops to 30%.

This overall effect of VAF on SV detection sensitivity is similar for different SV types, with one exception: VAF has little to no effect on FINS detection. This is unsurprising as FINS is difficult to detect for all SV callers even at 100% VAF (Figure 5). Interestingly, increasing VAF appears to negatively impact the precision of INS detection for Manta. This is due to the rapid increase in the absolute number of INS called by Manta with increasing VAF (10 INS at VAF = 0.05, 66 INS at VAF = 0.1 and 171 INS at VAF = 0.2), of which, many are FPs. The fluctuation in precision for INS detected by GRIDSS at varying VAF (Figure 9**B**) is due to the low numbers of total INS detected (only one INS at VAF ≤ 0.1, 12 INS at VAF = 0.2 and 22 INS at VAF = 1). Again, Delly behaves differently compared with other SV callers in that, there is more distinct variability in its performance for different SV types. In particular, Delly performs much better for DEL than other SV types, especially at VAF > 0.5.

As previously observed (Figure 6**)**, small SVs (50–100 bp) and large SVs (>1 Mbp) are more difficult to detect. However, the impact of VAF to detect different SV sizes is similar across the SV callers. The most notable difference is the counter-intuitive decrease in precision for Manta for detecting SV < 100 bp with increasing VAF (Figure 9**D**, Manta). Many of the FP SVs < 100 bp are INS events, corroborating with the similar trend observed above (Figure 9**B**, Manta), suggesting Manta has elevated FP calls for small INS especially with increasing VAF. Furthermore, many of the FP small INS appears to be a result of incorrect SV-type assignment by Manta; i.e. DUP and INV events reported as INS.

#### Deep sequencing is critical when VAF is <50%

**Figure 10:**
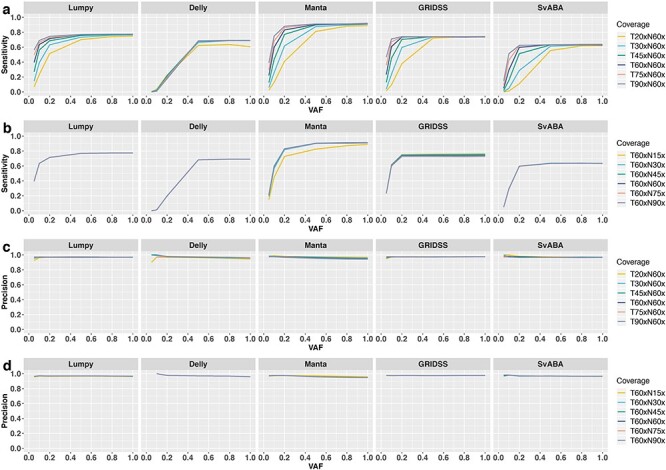
Joint impact of sequencing coverage and VAFs on SV detection. Shown are total sensitivity (**A**) and precision (**B**) across VAF with different sequencing coverage of the tumour samples (**A** and **C**) and match-normal samples (**B** and **D**), based on breakpoint precision threshold of 200 bp.

To determine if, and to what extent, increasing sequencing depth can improve SV detection at low VAF, we evaluated six levels of tumour (20×, 30×, 45×, 60×, 75× and 90×) and normal coverage (15×, 30×, 45×, 60×, 75× and 90×) at six VAF levels. As expected, increasing tumour sequencing coverage can greatly improve somatic SV detection sensitivity for samples with low VAF (Figure 10**A**, **B**). For example, at 0.2 VAF, increasing tumour coverage from 20× to 30× can increase sensitivity by 20% using Manta. However, the benefit of increasing coverage quickly saturates. For example, at 0.2 VAF, increasing tumour coverage from 60× to 90× yields only a 5% improvement in sensitivity for Manta. In contrast, the impact of tumour sequencing coverage has less impact for samples with higher VAF. For example, at 0.5 VAF, Manta sensitivity increases only by 6% from 20× to 30× and 1% from 60× to 90×. The absolute extent of impact from tumour sequencing coverage is different for different SV callers. In particular, Delly gains <10% sensitivity from 20× to 30× depth of tumour coverage and derives no further gain above 30×. It is notable that tumour coverage has almost no impact on the precision of SV detection regardless of VAF level (Figure 10**B**). The small fluctuation on Delly precision at low VAF is due to the small number of total detected SVs.

In contrast to tumour sequencing depth, sequencing coverage of the matched-normal sample has little impact on either sensitivity or precision, with the exception that increasing normal-sample coverage from 15× to 30× improves sensitivity for Manta (Figure 10**C**).

In summary, when VAF is high (>0.8), little value can be gained from increasing sequencing depth. In fact, when VAF is close to one, all evaluated SV callers have reasonable sensitivity (>60%) to identify somatic SVs at low sequencing coverage (20× to 30×). In contrast, deep tumour coverage (75× to 90×) is critical when VAF is low (<0.5). Finally, when VAF is <0.1, there is little to no power in SV discovery even for deeply sequenced (>90×) tumour samples.

### Impact of SegDup on SV detection

Genomic regions of low complexity, such as repeats and GC-rich regions [2], can result in ambiguous read-mapping, which can lead to incorrect read alignments and subsequent false variant detection. While different short-read aligners and their parameterizations can alleviate some of this issue, realignment is typically not performed for the detection of different variant types, thus placing the bulk of the work on variant detection tools. SV callers usually use a mapping quality (MAQ) threshold to ensure SVs are supported by unique mapping. Ambiguous mapping can also affect precise breakpoint prediction. In particular, SegDup, which comprise around 5% of the human genome, are sequences at different genomic loci that share a high level of (>90%) sequence identity [36], and is one of the biggest sources of false variant calls. In a recent study, it was found that germline SVs at low complexity and simple or short-tandem repeat regions have lower precision at different levels for all callers [9]. Here, we evaluate the impact of SegDup on somatic SV calling.

#### SegDup regions are prone to FP DUP and DEL calls

**Figure 11:**
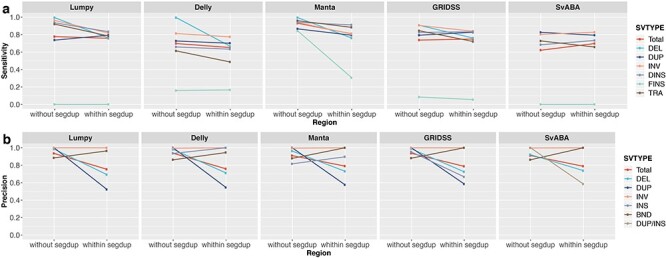
Sensitivity and precision of SV detection within and outside of segmental-duplicated regions. Results are based on simulation data with tumour and matched-normal coverage of 60×, VAF of 100% and breakpoints within 200 bp of simulated SVs.

On average, somatic SV residing within SegDups are 2.0% (1.9–12%) less likely to be detected and induce 15% (12–18%) higher FP calls than those outside of SegDup regions (Figure 11). The most significant impact on detection sensitivity was observed for FINS called by Manta and DELs from Delly. Unlike VAF and sequencing coverage, which affect sensitivity more than precision, the reverse is true for SegDups. Across all SV callers, precision is greatly reduced for SV collocating at SegDup regions, especially for DUP and DEL events; that is, FP DUP and DEL are mainly due to SegDup regions.

### Impact of sequencing biases on SV detection

It has been shown that GC-content bias (high or low GC content) can influence short-read sequencing depth of coverage [37], resulting in insufficient supporting reads for read-pair and split-read methods [19] and lower accuracy of assembly [38], which could in principal impact SV detection. Similarly, homopolymers are a known source of error for both sequencing and read-alignment, which can impact breakpoint resolution in SV detection. We formally tested these two biases on SV detection and found no significant association between GC bias at or flanking SV breakpoint and sensitivity (TP/FN) or precision (TP/FP) (Supplementary Table S2 available online at https://academic.oup.com/bib and Supplementary Data available online at https://academic.oup.com/bib). Similarly, we found no significant correlation between (1) the presence of a homopolymer, (2) the distance of a homopolymer to a breakpoint, (3) the length of the homopolymer or (4) the number of homopolymers around a SV breakpoint and SV detection sensitivity or precision (Supplementary Table S2 available online at https://academic.oup.com/bib and Supplementary Data available online at https://academic.oup.com/bib).

As many biases that impact sequencing can in turn affect MAQ, we also investigated the presence of association between MAQ and SV detection sensitivity and precision (Supplementary Data available online at https://academic.oup.com/bib). Statistically significant correlations were observed between MAQ and sensitivity (TP versus FN) for all SV callers and between MAQ and precision (TP versus FP) for Manta (Supplementary Table S2 available online at https://academic.oup.com/bib). As expected, most SegDup regions (60%) have lower MAQ (<40) due to ambiguous read-alignment, which explains the lower sensitivity and precision in detecting SVs around SegDup regions, as discussed above. In addition, FINS events consistently have lower MAQ (40–50) due to foreign sequences not having any matches in the reference genome. In sum, low MAQ explains the much lower recovery rate of FINS for all SV callers as well as the high FP rate for Manta.

## Concluding remarks

SVs are an important type of genomic alterations in cancer, but are intrinsically more difficult to detect than small variants from short-read NGS data. While the surge of new SV callers in the past few years has significantly enhanced our ability to detect SVs from genomics data, it has resulted in the unintended effect of ‘overchoice’. To overcome this, recent studies have attempted to compare the performance of a variety of SV callers, but these have focused predominantly on germline SVs and simple SV types [8, 9] and only on overall performance for somatic SVs [10]. In this review, we have added to this mounting evaluation effort by examining the effect of major factors affecting the ability of different methods in detecting somatic SVs. Although simulated data does not truly reflect the complexity of real cancer samples, the evaluation presented in this review provides an insight into the upper bound of what is achievable with current methodologies [9, 10].

Overall, we recapitulate previous observations that SV caller based on more than one method performs better than those relying on single methods [8, 9]. Furthermore, while SV callers based on the same detection method(s) have similar overall performance, they vary in their ability to detect different SV types and SV sizes. Therefore, for comprehensive detection of somatic SVs, it may still be necessary to use a combination of callers. From our evaluation, we found the pairwise union callsets of Manta and Lumpy or GRIDSS to provide the highest F1 value. However, it is worth noting that some SVs can give rise to copy number changes, which are not easily detected with SV callers alone. Read-depth methods are particularly suited for CNV detection, and potentially additionally needed, for the identification of SVs with associated copy number gains or losses (beyond a single copy as simulated in this study), especially for complex events [18, 39].

Moreover, we found that DELs tend to have a higher validation rate than other SV types. This likely explains the higher validation rate for DEL events [25]. However, we note that all SV callers have low discovery rate in calling novel sequence insertion (FINS) and relatively small SVs (<100 bp) and have high false discovery rate in calling large SVs (>1 Mbp) and SVs with highly homologous breakpoints. This is an area for improvement, either for future somatic SV callers for NGS data or for long-range sequencing technologies. For factors affecting SV detection, we have found VAF, due to tumour purity and/or intra-tumour heterogeneity, to have the biggest impact. As expected, sequencing coverage of the tumour sample can rescue some of the sensitivity lost; however, the amount of improvement is not linear and differs between SV callers. In general, little improvement is observed (<4%) beyond 60× depth of tumour coverage for VAF > 0.2 and there does not appear to be a significant impact from sequencing depth of coverage of the match-normal sample.

Additionally, and uniquely to this study, we evaluated SV callers ability in identifying inter-chromosomal rearrangements, such as DINS (copy-and-paste) and TRA (cut-and-paste) with inserted sequence from another chromosome. Due to inherent limitations of NGS data, inserted or translocated sequences cannot be fully captured. However, by jointly utilizing read-pairs, split-reads and local-assembly methods, BND of rearranged genomic fragments can be accurately resolved. We have shown that BND pairs of DINS and TRA are reported with high sensitivity and precision by SV callers that jointly use these methods (namely, Manta, GRIDSS and SvABA). Correct and precise identification of BNDs are important as they are essential for SV annotation. However, annotation of SV types remains challenging particularly for intra-chromosomal DINS and TRA, which often create misleading DEL and DUP signatures, and thus reported as such, even when there is no copy number gain and/or loss.

As the detection of small variants become routine and embedded within cancer genomic pipelines, it is imperative to start to consider and work towards capturing one of the most important genomic aberrations in cancer. In addition to the major factors evaluated in this review, other factors such as sequencing GC bias, insert library size, strand bias, MAQ and, of course, sequencing technology can also affect variant detection [10]—all of which should be considered. In this review, we examined the impact of GC-content bias and homopolymers and, based on our data, found no significant correlation with SV detection sensitivity and precision. In contrast, MAQ was found to be significantly correlated with SV detection performance, and these appear to be associated with SegDup regions and FINS events. As SV callers begin to saturate the bioinformatics field, it is timely to evaluate areas ripe for improvement. This review highlights these areas being the identification of FINS and detection of relatively small and very large SVs. Moreover, there is still the unattained goal of an SV caller capable of identifying all SV types and sizes and capable of capturing the full inserted or translocated sequences.

**Key Points**

- A comparison of seven most commonly used structural variant callers for cancer genomics was performed.
- Structural variant callers based on multiple detection methods are more sensitive and reproducible.
- Addition of a second SV caller to an already high-performing caller adds little value.
- Most SV callers have ~2 bp breakpoint resolution.
- Variant allele frequency has a logarithmic impact on sensitivity but little effect on precision.

## Supplementary Material

Supp_Methods_bbaa056

## Notes

**Tingting Gong** is a PhD candidate of The University of Sydney at Garvan Institute of Medical Research.

## Notes

**Vanessa M. Hayes** is a Petre Chair of Prostate Cancer at the The University of Sydney and Garvan Institute of Medical Research.

## Notes

**Eva K.F. Chan** was a Senior Bioinformatician at the Garvan Institute of Medical Research, and currently at NSW Health Pathology
